# Elevated mRNA expression of CHAC1 splicing variants is associated with poor outcome for breast and ovarian cancer patients

**DOI:** 10.1038/bjc.2011.510

**Published:** 2011-11-22

**Authors:** G Goebel, R Berger, A M Strasak, D Egle, E Müller-Holzner, S Schmidt, J Rainer, E Presul, W Parson, S Lang, A Jones, M Widschwendter, H Fiegl

**Affiliations:** 1 Department of Medical Statistics, Informatics and Health Economics, Innsbruck Medical University, Innsbruck A-6020, Austria; 2 Department of Gynaecology and Obstetrics, Innsbruck Medical University, Anichstraße 35, Innsbruck A-6020, Austria; 3 Department of Internal Medicine V, Innsbruck Medical University, Innsbruck A-6020, Austria; 4 Biocenter Innsbruck, Division of Molecular Pathophysiology, Innsbruck Medical University, Innsbruck A-6020, Austria; 5 Institute of Legal Medicine, Innsbruck Medical University, Innsbruck A-6020, Austria; 6 Department of Statistics, Faculty of Economics and Statistics, Leopold-Franzens University, Innsbruck A-6020, Austria; 7 Department of Gynaecological Oncology, UCL EGA Institute for Women's Health, University College London, London W1T 7DN, UK

**Keywords:** CHAC1, breast cancer, ovarian cancer, biomarker, prognosis

## Abstract

**Background:**

The role of CHAC1 (cation transport regulator-like protein 1), a recently identified component of the unfolded protein response (UPR) pathway, in gynaecological cancers has not yet been characterised. Now, this work illustrates CHAC1 mRNA expression and associated clinical outcome in breast and ovarian cancer.

**Methods:**

The prognostic value of CHAC1 and its two transcript variants was investigated in 116 breast and 133 ovarian tissues using quantitative real-time reverse-transcriptase PCR. Subsequently, we conducted functional studies using short-interfering RNA-mediated knockdown and plasmid-mediated overexpression of CHAC1 in breast and ovarian cancer cells.

**Results:**

Poorly differentiated tumours exhibited higher CHAC1 mRNA expression (breast cancer: *P*=0.004; ovarian cancer: *P*=0.024). Hormone receptor-negative breast tumours and advanced-staged ovarian cancers demonstrated elevated CHAC1 mRNA expression levels (*P*<0.001 and *P*=0.026, respectively). The multivariate survival analysis showed a prognostic value of both transcript variants in breast cancer (transcript variant 1: RR_death_ 6.7 (2.4–18.9); *P*<0.001), RR_relapse_ 6.7 (2.1–21.3); *P*=0.001); (transcript variant 2: RR_death_ 4.9 (2.0–12.4); *P*<0.001), RR_relapse_ 8.0 (2.4–26.8); *P*<0.001). Ovarian cancer patients aged younger than 62.6 years with high CHAC1 mRNA expression showed poorer relapse-free- and overall-survival (*P*=0.030 and *P*=0.012, respectively). In functional studies CHAC1 knockdown suppressed cell migration, whereas ectopic overexpression opposed these effects.

**Conclusion:**

High CHAC1 mRNA expression could be an independent indicator for elevated risk of cancer recurrence in breast and ovarian cancer.

Breast and ovarian cancer account for nearly one-third of all cancers in women worldwide (Ferlay *et al*, 2010). Although some progress has been made, these diseases still remain major causes of death in women. Further insight into the biology of these cancers is needed to improve patient outcome.

The unfolded protein response (UPR) pathway is a stress-signalling pathway in the endoplasmic reticulum. This signal transduction cascade is activated in a range of human solid tumours including breast cancer (Fernandez *et al*, 2000; Scriven *et al*, 2009). It has been shown that hypoxia and glucose deprivation factors, known to trigger the UPR pathway, enhance the metastatic potential and are linked to poor differentiation (Le *et al*, 2004). Endoplasmic reticulum stress initiated by the tumour microenvironment and by activation of the UPR is proposed to contribute to multiple disease features including apoptosis and tumour resolution, tumour dormancy, tumour growth, disease progression or even altered chemotherapeutic sensitivity (Scriven *et al*, 2007).

Many UPR components are overexpressed in human tumours such as breast tumours (Fernandez *et al*, 2000), hepatocellular carcinomas (Shuda *et al*, 2003), gastric tumours (Song *et al*, 2001) and oesophageal adenocarcinomas (Chen *et al*, 2001).

Recently CHAC1 a new UPR pathway member was identified. This molecule was discovered first in a co-regulated group of genes enriched for components of the ATF4 (activating transcription factor 4) arm of the UPR pathway (Gargalovic *et al*, 2006) and it was then characterised as a novel proapoptotic component of this pathway (Mungrue *et al*, 2009). Previously CHAC1, among other genes, was shown to be differentially expressed in pancreatic ductal adenocarcinoma in comparison with normal pancreatic ducts (Buchholz *et al*, 2005). Two alternatively spliced transcript variants of this molecule have been described until now. Transcript variant 1 (GenBank: NM_024111.3) represents the longer transcript and encodes the longer isoform a. Transcript variant 2 (GenBank: NM_001142776.1) lacks an alternate in-frame segment, compared with variant 1, resulting in a shorter protein (isoform b), compared with isoform a.

In this study we investigated the prognostic role of CHAC1 mRNA expression and its transcript variants in breast and ovarian cancer patients, and we describe the effect of CHAC1 on cell migration in breast and ovarian cancer to better understand its role in tumour biology and potential implication for cancer progression.

## Patients and methods

### General study design, patients and samples

We retrospectively studied CHAC1 mRNA expression by applying qRT–PCR to prospectively collected breast and ovarian tissue samples from patients with primary breast neoplasm treated at our Department (Department of Gynaecology and Obstetrics, Innsbruck Medical University, Austria). Samples have been collected during primary surgery. Univariate and multivariate survival models were used to explore the potential of CHAC1 mRNA expression in predicting relapse-free (RFS) and overall-survival (OS).

Furthermore, we re-examined the breast and ovarian cancer cases with respect to CHAC1 transcript variants 1 and 2 mRNA expression. Finally, we conducted functional studies using short-interfering RNA (siRNA)-mediated knockdown and plasmid-mediated overexpression of CHAC1 in breast cancer cells (Hs578T, BT-20) and ovarian cancer cells (HOC-7).

Clinical, pathological and follow-up data were stored in a database according to our hospital privacy rules. The patients were treated at our Department between February 1989 and March 2004. Written informed consent is not available from all patients. But in accordance with the Austrian law, the study was approved by the Ethics Committee of the Innsbruck Medical University (reference number: AM3634) and conducted in accordance with the Declaration of Helsinki Principles. Patient consent was not obtained under a waiver IRB approval. All samples were anonymised to guarantee the protection of privacy before performing the analysis. The study was performed in concordance with the Reporting Recommendations for Tumour Marker Prognostic Studies of the National Cancer Institute (McShane *et al*, 2005).

Frozen breast-tissue samples from 106 patients with primary breast cancer (aged 35.5–89.7 years; median age at diagnosis, 60.4 years) and 10 patients with benign breast diseases (aged 27.3–66.9 years, median age at diagnosis, 40.1 years), frozen ovarian-tissue samples from 103 patients with ovarian cancer (aged 24.1–87.1 years; median age at diagnosis, 62.6 years) and 30 patients with benign ovarian diseases (aged 38.2–85.8 years; median age at diagnosis, 55.7 years) were analysed. In ovarian cancer patients staging was performed in accordance with the International Federation of Gynaecology and Obstetrics (FIGO) classification system.

All patients were monitored within the outpatient follow-up programme of our Department.

The median observation period of the breast cancer patients was 7.5 years (0.9–17.0) and 4.13 years (0.09–20.0) for the ovarian cancer patients. No neoadjuvant chemotherapy was applied to the patients included in the study.

Overall, 23% of the breast cancer patients received only chemotherapy (*n*=24) and 37% only endocrine therapy (*n*=39); 25% received both chemotherapy and endocrine therapy (*n*=26); 6% received no adjuvant therapy (*n*=6); and 10% received only radiation therapy (*n*=11). Radiation therapy was applied in combination with chemotherapy or endocrine therapy in 55% (*n*=58). None of the patients received anti-HER2 therapy.

A platinum-based chemotherapy was part of the treatment for all but 12 ovarian cancer patients (8, 2 and 2 who had FIGO stage I, II and III, respectively). Clinicopathological features of all patients are summarised in Table 1.

Tumour specimens were obtained immediately after surgery, brought to our pathologist, a part of the tissue was pulverised under cooling with liquid nitrogen and stored at −70 °C. Oestrogen receptor (ER) and progesterone receptor (PR) status was identified by immunohistochemistry.

### Cell culture

The human breast cancer cell lines Hs578T and BT-20 were obtained from the American Type Culture Collection (ATCC, Manassas, VA, USA) and cells were cultured as recommended by the ATCC. Human ovarian carcinoma cell line HOC-7 was kindly provided by Dr C Dittrich (University of Vienna, Vienna, Austria) and cultured under standard conditions (Marth *et al*, 1997). Amplification of 15 STR loci and the gender-specific locus amelogenin was carried out in the Institute of Legal Medicine, Innsbruck Medical University, to authenticate these cell lines as described recently (Parson *et al*, 2005).

### CHAC1 knockdown cells

A human CHAC1-specific siRNA with no potential off-targets and a scrambled (scrbl) control were purchased from Qiagen (CHAC1: SI00642131; control: 1027280; Hilden, Germany).

Small-interfering RNA transfections were performed according to the manufacturer's protocol. All cell lines were transfected with 5 nmol l^−1^ siRNA against CHAC1 or control siRNA. Two days after transfection, the cells were treated further for *in-vitro* scratch assay or proliferation assay, respectively, and subsequently collected for western blots and real-time quantitative PCR analysis.

### CHAC1-overexpressing cells

We used CHAC1–pcDNA6 plasmid (CHAC1–V5eGFP construct) provided by the University of California at Los Angeles, USA. The control plasmid was produced by excision of the CHAC1 sequence using *Xba*1 and *Xho*1 (Fermentas, Leon-Rot, Germany), from the pcDNA6-V5eGFP backbone. Plasmid DNA was purified using miniprep and/or midiprep kits from Qiagen.

### *In vitro* scratch assay

The assay was performed as recently described (Liang *et al*, 2007; Berger *et al*, 2010). Untransfected cells (BT-20, Hs578T and HOC-7), CHAC1-knockdown cells, CHAC1 overexpressing and mock-treated cells were scratched by a pipette tip when cell confluence reached ∼90% and further cultivated for 24 h in fresh medium with reduced FCS amounts. The same fields were photographed immediately (0 h), and 4, 8 and 16 h later (Hs578T), or 24 and 48 h later (BT-20), or 14 and 48 h later (HOC-7) using a Kappa PS30 camera (Kappa opto-electronics GmbH, Gleichen, Germany). The experiments were repeated at least three times. Gap widths were measured using the free ImageJ software (http://rsbweb.nih.gov/ij/).

### Proliferation assay

Tumour cells were seeded in six-well plates at a density of 2 × 10^5^ or 2.5 × 10^5^ cells per ml (Hs578T), or 4 × 10^5^ (BT-20) or 3 × 10^5^ or 5.8 × 10^5^ (HOC-7) in Minimal Essential Medium (Gibco, Life technologies, Paisley, UK) with 10% FCS (PAA Laboratories GmbH, Pasching, Austria). Cells were trypsinised and counted (Beckman coulter and microscope counting chamber) after 48 or 72 h (Hs578T), or 48 or 96 h (BT-20) or 48 h (HOC-7).

### Apoptosis detection

Biotin-labeled POD TUNEL Apoptosis detection kit for adherent cell was used according to the manufacturer's protocol (GenScript, Piscataway, NJ, USA). Cells were counterstained with DAPI and analysed using an Olympus 1 × 70 inverted microscope (Olympus, Tokyo, Japan) in conjunction with Kappa ImageBase software V2.7.2.

Furthermore, cells were analysed by FACS analysis. Cells were incubated in propidium iodide buffer (50 *μ*g ml^−1^ propidium iodide, 0.1% Triton X-100, 0.1% trisodium citrate), for 4 h at 4 °C before FACS analysis.

### RNA isolation and mRNA expression analysis

Procedures were performed as previously described (Widschwendter *et al*, 2000; Mueller *et al*, 2003). Primers and probe for qRT-PCR for CHAC1 were purchased from Applied Biosystems (Foster City, CA, USA, Applied Biosystems Assay ID: Hs00899499_g1). Primers and probes for the TATA box-binding protein (endogenous RNA control) were used according to Bièche *et al* (1999). All reactions were checked if they are specific for mRNA and do not amplify genomic DNA.

Primers and probe for CHAC1 transcript variants: Transcript variant 1 (GenBank: NM_024111.3): forward: 5′-ATGCCTGGCCGTGTGG-3′, reverse: 5′-GCTTACCTGCTCCCCTTGC-3′, TaqMan probe: 5′-FAM-CAGCCCTCATGATCTTCAAGGAGCGT-TAMRA-3′ Transcript variant 2 (GenBank: NM_001142776.1): forward: 5′-GGTTCTGCTCCCCTTGCA-3′, reverse: 5′-CGTGTGGTGACGCTCCTTG-3′, TaqMan probe: 5′-FAM-CCCAAGTGCAGCCCTCATGA-TAMRA-3′.

### Western blot analysis

Western blot analysis was performed as previously described (Berger *et al*, 2010). The following antibodies were used: CHAC1 (1 : 250; Sigma-Aldrich, St Louis, MO, USA), *β*-actin (1 : 1000; Abcam, Cambridge, UK) and glyceraldehyd-3-phosphate dehydrogenase (1 : 10 000; Biomol, Hamburg, Germany).

### Statistical analysis

Descriptive results are presented as median and interquartile range. For the comparison of CHAC1 expression between groups, a two-sided *t*-test, or in case of comparisons between more than two groups, ANOVA was applied to the log-transformed values of CHAC1 expression. The correlation between the log-transformed CHAC1 expression values was analysed using the Pearson's correlation coefficient. For survival analysis, the CHAC1 mRNA expression was dichotomised into low and high using the median expression value.

Relapse-free survival was defined as the time from surgery to histopathological confirmation of distant metastases or regional recurrence. Overall survival was defined as the time from surgery to death from any cause or to the last clinical inspection. To estimate hazard ratios with 95% confidence intervals, we first calculated univariate Kaplan–Meier curves for dichotomised age, tumour size, stage, grade, chemotherapy and CHAC1 mRNA expression using the log-rank test to compare the survival distributions between groups. For breast cancer specimens additionally menopausal and lymph node status, hormone receptor (HR) and HER-2/neu status and the application of endocrine and radiation therapies were considered.

A time-independent Cox proportional hazard approach was used for multivariate survival analysis using all variables of the univariate analysis (Table 2). Furthermore, a backward conditional stepwise variable selection procedure was used, with *P*<0.05 for entering and *P*>0.1 for removing a variable in the model. For both multivariate models dichotomised CHAC1, CHAC1 transcript variants 1 and 2 mRNA expression values were used as exposure variables.

In sensitivity analysis we further aimed to flexibly investigate the effect of CHAC1 and CHAC1 transcript variants 1 and 2 on the above endpoints, including CHAC1 as a log-transformed continuous variable in univariate and multivariate models, using penalised splines (P-splines) in extended, restricted maximum-likelihood optimal Cox-type additive hazard regression (Strasak *et al*, 2009). Data points of the 10–90% interpercentile range of CHAC1 were used for the calculation of Supplementary Figure 1. *P*-values less than 0.05 were considered as statistically significant. SPSS 18.0 (SPSS Inc., Chicago, IL, USA), STATA/MP 10.0 (StataCorp., LP, College Station, TX, USA) and BayesX 1.51 (Department of Statistics of the Ludwig-Maximilians-University Munich, Munich, Germany) were used for the statistical analyses.

## Results

### CHAC1 mRNA expression and clinical outcomes in breast cancer

**Figure 1:**
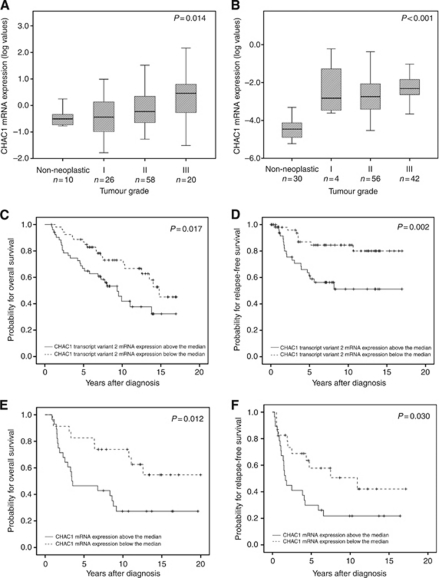
CHAC1 mRNA expression in tissue samples. (**A**) Non-neoplastic (NN) and neoplastic grade I–III breast cancer samples. (**B**) Non-neoplastic and neoplastic grade I–III ovarian cancer samples. Outliers and extreme values are excluded. CHAC1 transcript variant 2 mRNA expression and (**C**) overall and (**D**) relapse-free survival analyses in 106 breast cancer patients. CHAC1 mRNA expression and (**E**) OS and (**F**) RFS analysis in 51 younger ovarian cancer patients (age <median age of 62.6 years).

We found an increase of CHAC1 mRNA expression from benign neoplastic tissues through to grade III cancer tissues (*P*=0.014; Figure 1A). Clinicopathological analysis of cancer tissues provided a similar result (*P*=0.004; Table 1). Increased CHAC1 mRNA expression was found in tumours lacking ER (*P*<0.001) and PR (*P*<0.001; Table 1a). Furthermore, we identified a positive association with tumour size (*P*=0.011; Table 1a).

Univariate survival analysis of all 106 breast cancer patients revealed no significant association of total CHAC1 mRNA expression with the clinical endpoints (Table 2a), whereas high total CHAC1 mRNA expression was strongly associated with a high risk of death and relapse in the multivariate Cox regression model (RR_death_ (1.3–7.1); *P*=0.012, RR_relapse_ 4.8 (1.6–14.6); *P*=0.005) (Table 3). CHAC1 also remained as strongest independent factor in the backward Cox regression model (RR_death_ 2.9 (1.3–6.4); *P*=0.008, RR_relapse_ 5.2 (1.8–15.2); *P*=0.002).

### CHAC1 transcript variants 1 and 2 mRNA expression

The mRNA expression values of total CHAC1 strongly correlated with the CHAC1 transcript variants 1 and 2 in breast cancer (*r*=0.94; *P*<0.001 and *r*=0.97; *P*<0.001). Expression of transcript variant 2 was significantly associated with poor outcome for OS (*P*=0.02; Table 2a) and with a poor RFS (*P*<0.01; Table 2a) in univariate survival analysis. The Kaplan–Meier survival curves for transcript variant 2 are shown in Figures 1C and D.

When adjusting for clinicopathological factors and therapies, the multivariate survival analysis showed significant prognostic value of transcript variant 1 (RR_death_ 6.7 (2.4–18.9); *P*<0.001), RR_relapse_ 6.7 (2.1–21.3); *P*=0.001) and transcript variant 2 (RR_death_ 4.9 (2.0–12.4); *P*<0.001), RR_relapse_ 8.0 (2.4–26.8); *P*<0.001) (Table 3). Backward selection of variables confirmed the full model for transcript variant 1 (RR_death_ 4.3 (1.9–9.8); *P*<0.001), RR_relapse_ 8.2 (2.7–24.9); *P*<0.001) and transcript variant 2 (RR_death_ 4.0 (1.8–9.0); *P*=0.001), RR_relapse_ 7.6 (2.5–23.0); *P*<0.001).

### Statistical validation of survival associations in breast cancer

Validating the association of CHAC1 and its transcript variants with survival within breast-cancer entities, a P-spline regression model confirmed the results of the main multivariate Cox model in sensitivity analysis (Supplementary Figure 1).

### CHAC1 mRNA expression and clinical outcomes in ovarian cancer

To analyse whether an aberrant CHAC1 mRNA expression is also associated with poor outcomes in other female malignancies, we analysed 103 ovarian cancer tissues and 30 normal ovarian specimens. The mRNA expression values of total CHAC1 strongly correlated with the CHAC1 transcript variants 1 and 2 also in ovarian cancer (*r*=0.88; *P*<0.001 and *r*=0.96; *P*<0.001).

We observed a significant increasing trend of CHAC1 mRNA expression from non-neoplastic tissues to grade III neoplastic tissues (*P*<0.001; Figure 1B). Again, poorly differentiated cancers demonstrated higher CHAC1 levels (*P*=0.024; Table 1b). A high CHAC1 mRNA expression was also associated with advanced tumour stage (*P*=0.026; Table 1b).

Univariate analysis of 103 ovarian cancer patients revealed prognostic significance for CHAC1 mRNA expression only in younger patients (<median age of 62.3 years) for OS (*P*=0.012; Table 2b, Figure 1E) and RFS (*P*=0.03; Table 2b, Figure 1F). The same findings were revealed for premenopausal women (data not shown).

In ovarian cancer, the multivariate survival analysis showed no significant prognostic value of CHAC1 (data not shown).

### CHAC1 influences cell migration and proliferation

Owing to the identified associations of CHAC1 mRNA expression and clinical outcome data in breast and in ovarian cancer patients, we were interested in functional effects of CHAC1 knockdown and CHAC1 overexpression in cancer cells.

Hence, we measured cell migration by means of an *in vitro* scratch assay and proliferation in Hs578T and BT-20 breast cancer and HOC-7 ovarian cancer wild-type cells, CHAC1 knockdown cells and cells treated with a scrbl siRNA as negative control.

**Figure 2:**
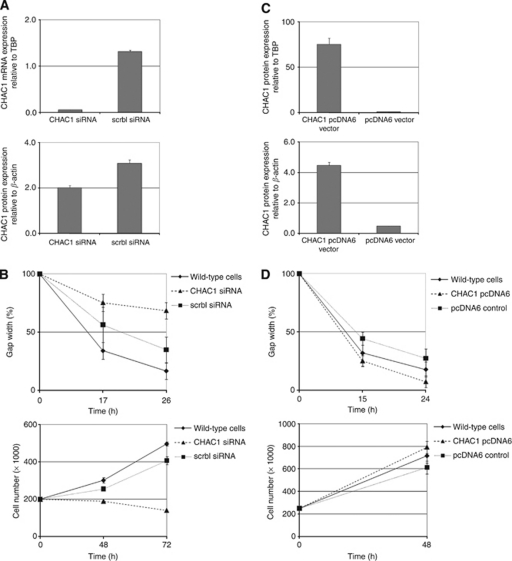
CHAC1 knockdown and overexpression analysis in Hs578T cells. Results of at least three independent experiments are shown. (**A**) CHAC1 mRNA and protein downregulation after treatment with siRNA. (**B**) *In vitro* scratch assay and proliferation analysis of wild-type breast cancer cells, CHAC1 knockdown cells (CHAC1 siRNA) and mock-transfected cells (scrambled (scrbl) siRNA) cells. (**C**) CHAC1 mRNA and protein overexpression after transfection with CHAC1–pcDNA6 or the pcDNA6 control vector. (**D**) *In vitro* scratch assay and proliferation analysis of wild-type breast cancer cells, CHAC1-overexpressing cells (CHAC1–pcDNA6) and mock-transfected cells (pcDNA6) cells. Results of scratch assays were plotted as percentage of wound closure relative to hour 0. TBP, TATA box-binding protein.

In Hs578T breast cancer cells a 96% or 35% CHAC1 knockdown was revealed at the mRNA level or the protein-level, respectively, in comparison with scrbl siRNA-treated cells (Figure 2A). We identified a significantly reduced migration and proliferation in CHAC1 knockdown cells (Figure 2B). Apoptosis measurements by means of FACS analysis or TUNEL staining, respectively, showed no differences between knockdown and control cells (data not shown).

Next, we performed again an *in vitro* scratch assay to measure cell migration of CHAC1-overexpressing Hs578T cells and cells transfected with a control plasmid as negative control. In comparison with the control cells a 83-fold or 10-fold increase was revealed in CHAC1 mRNA or protein expression, respectively, in Hs578T cells (Figure 2C). We identified significantly increased migration and proliferation in CHAC1-overexpressing cells in comparison with mock-treated cells, respectively (Figure 2D), but no effect on apoptosis (data not shown).

In BT-20 breast cancer cells, a 56% CHAC1 knockdown was revealed at the protein level (Supplementary Figure S2A). In this cell line only a tendency of a reduced migration and proliferation was observed (Supplementary Figure S2B).

In CHAC1-overexpressing BT-20 cells (1.5-fold increase in protein expression; Supplementary Figure S2C), we identified an increased migration and only a tendency of an increased proliferation (Supplementary Figure S2D).

To elucidate the role of CHAC1 in ovarian cancer we analysed CHAC1 knockdown and overexpression in HOC-7 ovarian cancer cells. In HOC-7 cells, a 21% CHAC1 knockdown was revealed at the protein level (Supplementary Figure S3A). Again, we identified a reduced migration in the knockdown cells in comparison with the control cells without affecting proliferation (Supplementary Figure S3B). In CHAC1-overexpressing HOC-7 cells (1.3-fold increase in protein expression; Supplementary Figure S3C) an increased migration was observed without affecting proliferation (Supplementary Figure S3D).

## Discussion

This is the first pilot study, which shows an association of CHAC1 mRNA expression in tumour tissues with the survival of breast and ovarian cancer patients. CHAC1 has been identified as a novel proapoptotic component of the UPR pathway, which itself responds to endoplasmic reticulum stress (Gargalovic *et al*, 2006; Mungrue *et al*, 2009).

We identified a positive correlation between poor tumour differentiation and higher CHAC1 mRNA expression levels in breast and ovarian cancer. Recently it was described in human parthenogenetic-induced pluripotent stem cells that CHAC1 is negatively regulated by miRNA-370, which is an imprinted miRNA (Stelzer *et al*, 2011). It was suggested that distinct regulatory imprinted small RNAs such as miRNA-370 and their targets such as CHAC1 have substantial roles in cellular differentiation (Stelzer *et al*, 2011).

Moreover, we found that a high mRNA expression of CHAC1 or related transcript variants were an independent poor prognostic marker for outcome in breast cancer patients. In the present study, the overall 5-year survival rate of breast cancer patients with low CHAC1 expression was 49% compared with only 35% for patients with high CHAC1 expression. Although this difference appears substantial when inspecting the Kaplan–Meier curves, it only reached statistical significance for the expression of both transcript variants in the univariate survival model for RFS (74% *vs* 53% and 78% *vs* 49%, respectively) and only for transcript variant 2 in OS (50 *vs* 34%). Considering the significant association of CHAC1 expression in breast cancer samples with most of the clinicopathological features (and their role as confounders), an inclusion of the features in the multivariate model showed a consistent strong independent prognostic role of CHAC1 and its splicing variants for RFS and OS, which was confirmed by additional sensitivity analyses. This finding is also supported by the significant correlation of CHAC1 expression and CHAC1 transcript variants. As mentioned above, on the basis of the increased expression of CHAC1 in HR-negative breast tumours, a stratified multivariate subgroup analysis might provide more insight and address the question, if the prognostic value of CHAC1 differs between patients with poorly differentiated or aggressive tumour cells and tumour entities with presence of HR on their surface. As a limitation, our case number did not provide this possibility. Furthermore it should be considered that the case–control design hinders the establishment of a causal relationship between elevated CHAC1 and poor outcome.

In ovarian cancer we observed only in younger patients (age <median age of 62.6 years as well as premenopausal women) an association between high CHAC1 mRNA expression levels and poor OS and RFS. The prognostic significance of CHAC1 mRNA expression determined specifically for younger women could provide indication that strong CHAC1 mRNA expression may be associated with an earlier development of more aggressive tumours.

Owing to the associations identified between high CHAC1 mRNA expression levels and poor survival, especially with RFS, in breast and partly in ovarian cancer patients, we hypothesised that CHAC1 may have a role in cell migration and proliferation. We found a significantly reduced migration and proliferation *in vitro*, in CHAC1 knockdown Hs578T breast cancer cells and, conversely, we witnessed increased migration and proliferation in CHAC1-overexpressing cells. But in CHAC1 knockdown BT-20 cells only a tendency of a reduced migration *in vitro* was observed, whereas in CHAC1 overexpression BT-20 cells an increased migration was identified.

In CHAC1-knockdown HOC-7 ovarian cancer cells we found a significantly reduced migration and conversely a tendency of an increased migration in CHAC1-overexpressing cells.

Recently, CHAC1 was described as a novel proapoptotic component in human aortic endothelial cells and in human embryonic kidney cells (HEK 293; Mungrue *et al*, 2009). However, in our CHAC1-knockdown and overexpression experiments no association with apoptosis was observed. It is known that the UPR pathway activation in cancer might result in apoptosis and disease resolution, or an anti-apoptotic, pro-angiogenic drive, resulting in disease progression (Ma and Hendershot, 2004; Scriven *et al*, 2007). Hence, it remains unclear how UPR activation in solid tumours balances cell survival and cell death.

As UPR is suspected to be responsible for the failure of some patients to respond to chemotherapy, it could provide a target for improving existing treatments or the discovery of new anti-cancer targets (Scriven *et al*, 2009). Additional studies will improve the understanding of the link between CHAC1 and patient resistance to breast cancer therapies.

In summary, our data show that CHAC1 correlated with tumour differentiation and survival in breast and partly in ovarian cancer. Cell migration was revealed to be in part affected by CHAC1 expression. Owing to the limitations of this pilot study, further studies should elucidate the role of CHAC1 and its transcript variants as potential biomarkers for identifying patients with high risk of cancer recurrence.

## Figures and Tables

**Table 1:** Association of CHAC1 mRNA expression with clinicopathological features. (a) 106 Primary breast cancer patients; (b) 103 primary ovarian cancer patients

		**CHAC1 mRNA expression logarithmic values (normal to TBP)**	
	***n***	**Mean (±s.d.)**	***P*-value**
*(a)*			
*Size*			
T1	33	−0.41 (0.59)	0.011
T2/3/4	73	−0.01 (0.82)	
			
*LN*			
Negative	44	−0.17 (0.72)	NS
Positive	56	−0.15 (0.80)	
NA	6		
			
*Tumour grade*			
I	26	−0.41 (0.76)	0.004
II	58	−0.14 (0.67)	
III	20	0.35 (0.93)	
NA	2		
			
*MP*			
Premenopausal	20	−0.35 (0.76)	NS
Postmenopausal	86	−0.07 (0.78)	
			
*HER2*			
Score 0/+	53	0.01 (0.89)	NS
Score ++/+++	34	−0.22 (0.63)	
NA	19		
			
*ER*			
Negative	37	0.38 (0.73)	<0.001
Positive	69	−0.39 (0.67)	
			
*PR*			
Negative	41	0.40 (0.76)	<0.001
Positive	65	−0.45 (0.59)	
			
*HR*			
Negative	34	0.46 (0.68)	<0.001
Positive	72	−0.39 (0.67)	
			
*(b)*			
*MP*			
Premenopausal	23	−2.6 (1.0)	NS
Postmenopausal	80	−2.5 (1.0)	
			
*Tumour stage*			
I/II	27	−2.8 (1.1)	0.026
III/IV	76	−2.4 (0.9)	
			
*Tumour grade*			
I/II	60	−2.6 (1.1)	0.024
III	42	−2.2 (0.8)	
NA	1		
			
*Histological type*			
Serous	45	−2.5 (0.9)	NS
Mucinous	32	−2.7 (1.0)	
Endometrioid	16	−2.0 (0.9)	
Clear cell	10	−2.5 (1.0)	

Abbreviations: CHAC1=cation transport regulator-like protein 1; HER2=human epidermal growth factor receptor 2 status; HR=hormone receptor status; ER=oestrogen receptor status; LN=lymph node status; MP=menopausal status; NA=not available; NS=not significant; PR, progesterone receptor status; TBP=TATA box-binding protein.

**Table 2:** Univariate survival analysis. (a) Overall survival and relapse free survival in 106 patients with primary breast cancer; (b) Overall survival and relapse-free survival in 103 ovarian cancer patients

	**Overall Survival**		**Relapse-free survival**	
**Variable**	**No. of patients (died/total)**	***P*-value (log-rank test)**	**No. of patients (relapsed/total)**	***P*-value (log-rank test)**
(*a*)				
*Size*				
T1	8/33	0.037	6/33	0.138
T2/3/4	39/73		22/73	
				
*LN*				
Negative	13/44	0.016	6/44	0.007
Positive	31/56		22/56	
				
*Tumour grade*				
I	14/26	0.340	5/26	0.627
II	26/58		18/58	
III	7/20		5/20	
				
*MP*				
Premenopausal	5/20	0.039	5/20	0.507
Postmenopausal	42/86		23/86	
				
*HER2*				
Negative	25/53	0.300	13/53	0.605
Positive	11/34		8/34	
				
*ER*				
Negative	15/37	0.330	11/37	0.866
Positive	32/69		17/69	
				
*PR*				
Negative	18/41	0.933	12/41	0.494
Positive	29/65		16/65	
				
*HR*				
Negative	14/34	0.460	10/34	0.863
Positive	33/72		18/72	
				
*Chemotherapy*				
No	24/56	0.633	9/56	0.037
Yes	23/50		19/50	
				
*Radiation therapy*				
No	18/36	0.309	5/36	0.139
Yes	29/69		23/69	
				
*Endocrine therapy*				
No	17/41	0.363	10/41	0.639
Yes	30/65		18/65	
				
*CHAC1 mRNA expression*				
Low (<median)	20/53	0.089	10/53	0.051
High (>median)	27/53		18/53	
				
*CHAC1 transcript variant 1 mRNA expression*				
Low (<median)	19/53	0.076	9/53	0.033
High (>median)	28/53		19/53	
				
*CHAC1 transcript variant 2 mRNA expression*				
Low (<median)	19/53	0.017	8/53	0.002
High (>median)	28/53		20/53	
				
				
(*b*)				
*Age*				
<62.6 years	29/51	0.002	32/51	0.604
⩾62.6 years	42/52		27/52	
				
*Tumor stage*				
I/II	14/27	0.024	8/27	0.005
III/IV	57/76		51/76	
				
*Tumor grade*				
I/II	25/60	0.001	25/60	0.001
III	34/42		34/42	
				
*Chemotherapy*				
no	8/12	0.757	4/12	0.679
yes	63/91		55/91	
				
*CHAC1 mRNA expression*				
Low (<median)	32/51	0.227	24/51	0.069
High (⩾median)	39/52		35/52	
				
*Age subgroup analysis (Age <median age)*				
Low CHAC1 expression	9/23	0.012	11/23	0.030
High CHAC1 expression	20/28		21/28	
				
*CHAC1 transcript variant 1 mRNA expression*				
Low (<median)	32/50	0.315	25/50	0.165
High (⩾median)	39/51		34/51	
				
*Age Subgroup Analysis (Age <median age)*				
Low CHAC1 TV1 expression	9/23	0.017	12/23	0.121
High CHAC1 TV1 expression	20/28		20/28	
				
*CHAC1 transcript variant 2 mRNA expression*				
Low (<median)	32/50	0.227	25/50	0.101
High (⩾median)	39/51		34/51	
				
*Age subgroup analysis (Age <median age)*				
Low CHAC1 TV2 expression	10/24	0.030	13/24	0.158
High CHAC1 TV2 expression	19/27		19/27	

Abbreviations: CHAC1 = cation transport regulator-like protein 1; ER = oestrogen receptor status; HER2 = human epidermal growth factor receptor 2 status; HR = hormone receptor status, LN = lymph node status; MP = menopausal status; PR = progesterone receptor status; TV1 = transcript variant 1; TV2 = transcript variant 2.

**Table 3:** Multivariate Cox regression survival analysis of 106 patients with primary breast cancer. (a) Overall survival; (b) relapse-free survival

	**Overall survival**							
	**Regression model without CHAC1 mRNA expression**		**Regression model including total CHAC1 mRNA expression**		**Regression model including CHAC1 transcript variant 1 mRNA expression**		**Regression model including CHAC1 transcript variant 2 mRNA expression**	
**Variable**	**RR of death (95% CI)**	***P*-value**	**RR of death (95% CI)**	***P*-value**	**RR of death (95% CI)**	***P*-value**	**RR of death (95% CI)**	***P*-value**
*(a)*								
*Age*								
<Median age	2.9 (1.1–7.9)	0.031	3.4 (1.2–9.3)	0.020	2.9 (1.1–8.0)	0.038	3.6 (1.4–9.7)	0.009
>Median age								
								
*Size*								
T1	2.6 (0.9–7.3)	0.071	3.1 (1.0–9.0)	0.043	3.7 (1.2–11.0)	0.021	3.2 (1.1–9.3)	0.028
T2/3/4								
								
*LN*								
Negative	1.7 (0.7–4.1)	0.268	1.6 (0.7–4.1)	0.293	2.5 (0.9–6.7)	0.073	2.0 (0.8–5.2)	0.163
Positive								
								
*Tumour grade*								
I	1.0 (0.6–1.7)	0.986	1.1 (0.6–1.8)	0.779	1.0 (0.6–1.8)	0.938	1.0 (0.6–1.7)	0.913
II								
III								
								
*MP*								
Premenopausal	1.2 (0.4–4.1)	0.752	1.0 (0.3–3.4)	0.977	1.4 (0.4–4.7)	0.608	0.7 (0.2–2.5)	0.616
Postmenopausal								
								
*HER2*								
Negative	1.2 (0.5–2.7)	0.724	1.3 (0.6–3.2)	0.514	1.3 (0.5–3.1)	0.581	1.3 (0.5–2.9)	0.577
Positive								
								
*HR*								
Negative	2.7 (0.7–10.7)	0.149	5.5 (1.2–26.2)	0.031	13.0 (2.3–73.2)	0.004	4.2 (1.0–17.3)	0.045
Positive								
								
*Chemotherapy*								
No	1.0 (0.4–2.6)	0.959	0.8 (0.3–1.9)	0.564	0.5 (0.2–1.3)	0.154	0.5 (0.2–1.4)	0.213
Yes								
								
*Radiation therapy*								
No	1.3 (0.6–3.0)	0.509	1.4 (0.6–3.2)	0.414	0.9 (0.4–2.2)	0.894	1.2 (0.5–2.9)	0.603
Yes								
								
*Endocrine therapy*								
No	0.6 (0.2–2.1)	0.472	0.6 (0.2–2.1)	0.408	0.4 (0.1–1.3)	0.129	0.9 (0.3–2.9)	0.821
Yes								
								
*CHAC1 mRNA expression (total or transcript variants, respectively)*								
Low (⩽median)			3.0 (1.3–7.1)	0.012	6.7 (2.4–18.9)	<0.001	4.9 (2.0–12.4)	<0.001
High (>median)								
								
*(b)*								
Age (in years)	3.5 (1.0–12.3)	0.050	3.0 (0.9–10.9)	0.087	2.9 (0.8–11.1)	0.112	3.8 (1.0–13.9)	0.044
<Median age								
>Median age								
								
*Size*								
T1	1.5 (0.4–5.6)	0.523	2.1 (0.5–8.8)	0.310	2.2 (0.5–9.5)	0.289	2.5 (0.6–11.2)	0.218
T2/3/4								
								
*LN*								
Negative	1.9 (0.6–6.5)	0.283	2.2 (0.6–7.6)	0.222	2.6 (0.7–9.4)	0.143	2.1 (0.6–7.7)	0.257
Positive								
								
*Tumour grade*								
I	0.9 (0.5–1.7)	0.736	0.9 (0.4–1.9)	0.802	0.9 (0.4–1.8)	0.687	0.8 (0.4–1.6)	0.512
II								
III								
								
*MP*								
Premenopausal	0.8 (0.2–2.9)	0.713	0.6 (0.2–2.3)	0.462	0.7 (0.2–2.8)	0.624	0.4 (0.1–1.7)	0.221
Postmenopausal								
								
*HER2*								
Negative	1.2 (0.4–3.4)	0.786	1.2 (0.4–3.8)	0.729	1.3 (0.4–4.2)	0.678	1.6 (0.5–5.2)	0.462
Positive								
								
*HR*								
Negative	2.4 (0.4–14.1)	0.349	4.5 (0.6–33.4)	0.139	6.5 (0.8–55.0)	0.084	4.2 (0.6–32.2)	0.163
Positive								
								
*Chemotherapy*								
No	2.4 (0.7–8.9)	0.180	1.4 (0.4–5.0)	0.631	1.2 (0.3–4.6)	0.760	1.0 (0.3–3.7)	0.955
Yes								
								
*Radiation therapy*								
No	3.6 (1.0–13.8)	0.056	3.5 (0.9–13.2)	0.065	2.9 (0.8–11.4)	0.122	4.0 (1.0–16.0)	0.052
Yes								
								
*Endocrine therapy*								
No	0.8 (0.2–4.3)	0.817	0.7 (0.1–4.2)	0.739	0.6 (0.1–3.4)	0.525	0.7 (0.1–4.6)	0.741
Yes								
								
*CHAC1 mRNA expression (total or transcript variants, respectively)*								
Low (⩽median)			4.8 (1.6–14.6)	0.005	6.7 (2.1–21.3)	0.001	8.0 (2.4–26.8)	<0.001
High (>median)								

Abbreviations: CHAC1=cation transport regulator-like protein 1; HER2=human epidermal growth factor receptor 2 status; HR=hormone receptor status; LN=lymph node status; MP=menopausal status; RR=relative risk.
