# Supplementary figures and images for: Elevated mRNA expression of CHAC1 splicing variants is associated with poor outcome for breast and ovarian cancer patients
Source: Br J Cancer. 2011 Nov 22;106(1):189–98. doi: 10.1038/bjc.2011.510 (PMC3251857; doi:10.1038/bjc.2011.510)

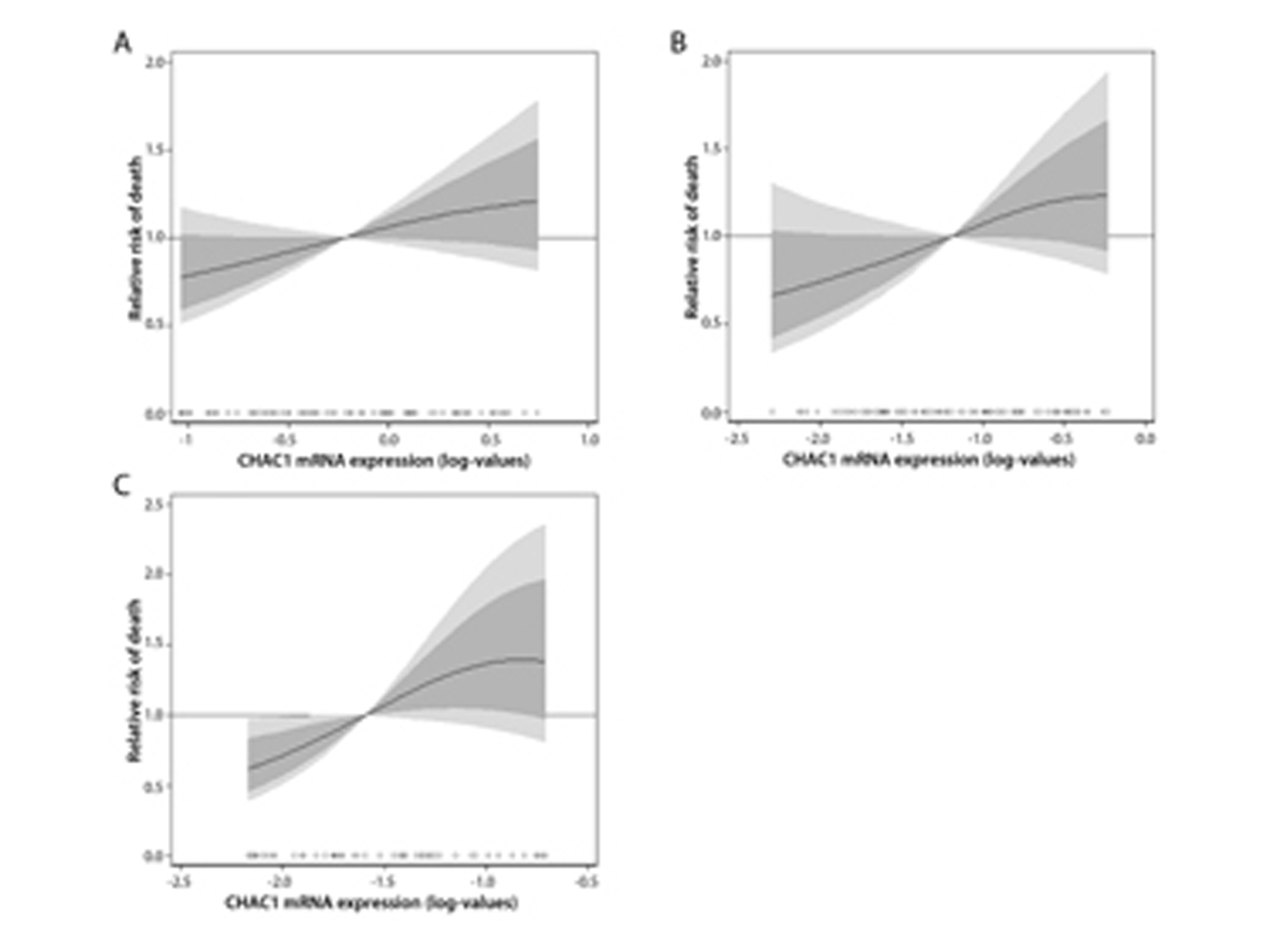

Supplement: Supplementary Figure S1 [file bjc2011510x1.tif]

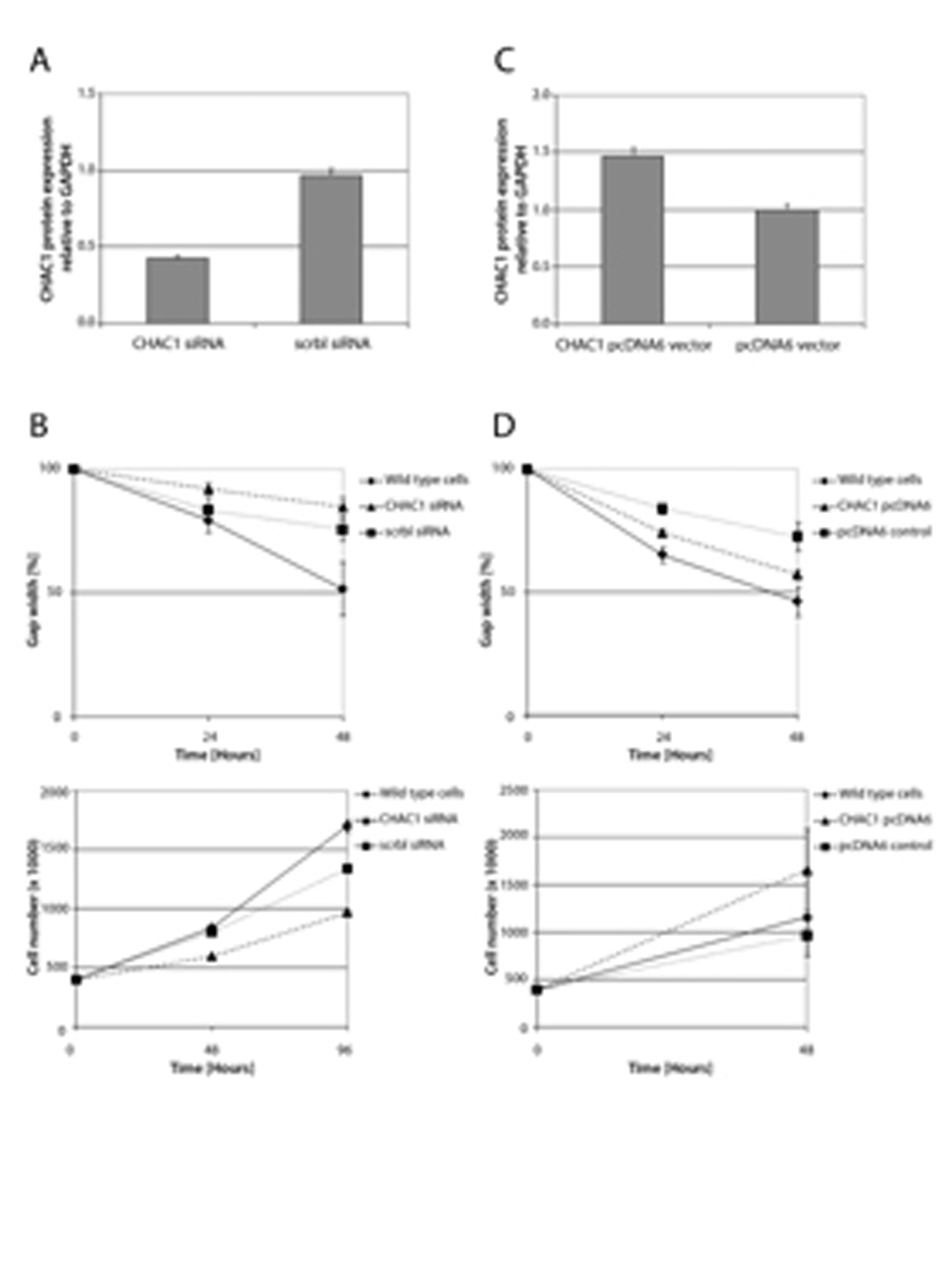

Supplement: Supplementary Figure S2 [file bjc2011510x2.tif]

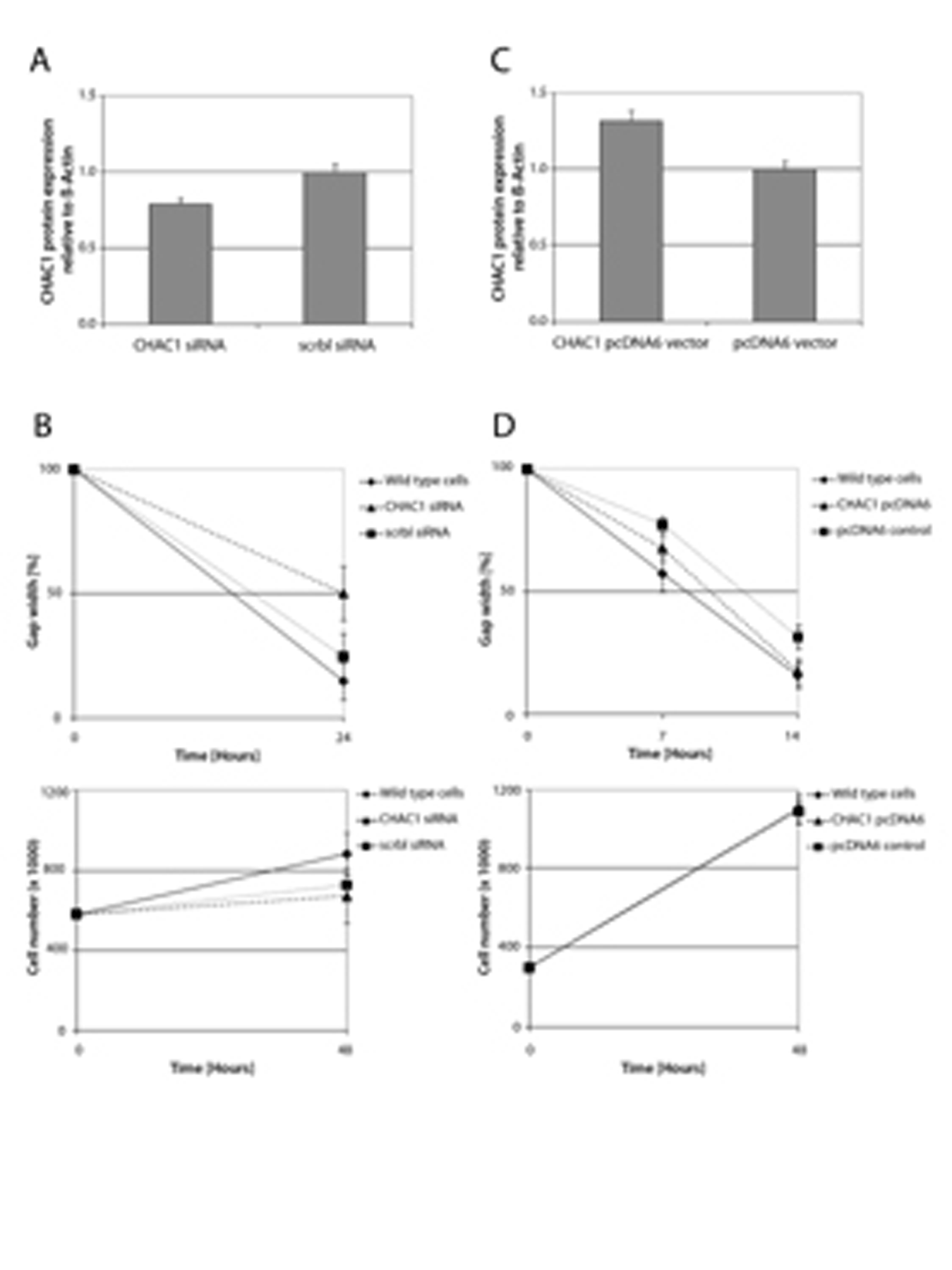

Supplement: Supplementary Figure S3 [file bjc2011510x3.tif]
