# Supplementary material for: Elevated mRNA expression of CHAC1 splicing variants is associated with poor outcome for breast and ovarian cancer patients
Source: Br J Cancer. 2011 Nov 22;106(1):189–98. doi: 10.1038/bjc.2011.510 (PMC3251857; doi:10.1038/bjc.2011.510)
Supplement: Supplementary Figure Legends [file bjc2011510x4.doc]

**Description of additional data files**

**Supplementary Figure S1:** **Penalized splines sensitivity analysis.** Estimated adjusted hazard ratios (solid lines) with 80% (dark grey) confidence intervals for the association of **(A)** CHAC1, **(B)** CHAC1 transcript-variant 1 and **(C)** CHAC1 transcript-variant 2 mRNA Expression, analyzed as continuous variable, with mortality in breast cancer patients from REML-optimal extended Cox-type additive hazard regression. CHAC1 mRNA expression was adjusted for age and tumor staging. The effect of CHAC1 mRNA expression on risk of the above end-points was modeled with a P-spline expansion of CHAC1. The median of CHAC1 mRNA Expression was used as reference value for the calculation of hazard ratios. The x–Axis is labeled by original CHAC1 mRNA expression values, whereas the y-Axis shows the adjusted hazard ratio.

**Supplementary Figure S2: CHAC1 knock-down and overexpression analysis in BT-20 breast cancer cells.** Results of at least three experiments are shown. **(A)** CHAC1 protein down-regulation after treatment with siRNA. **(B)** *In-vitro* Scratch Assay and Proliferation analysis of Wild-type breast cancer cells, CHAC1 knock-down cells (CHAC1-siRNA) and mock-transfected cells (scrbl-siRNA) cells. **(C)** CHAC1 protein overexpression after transfection with CHAC1-pcDNA6 or the pcDNA6 control vector. **(D)** *In-vitro* Scratch Assay and Proliferation analysis of Wild-type breast cancer cells, CHAC1 overexpressing cells (CHAC1-pcDNA6) and mock-transfected cells (pcDNA6) cells. Results of scratch assays were plotted as percentage of wound closure relative to hour 0.

**Supplementary Figure S3: CHAC1 knock-down and overexpression analysis in HOC7 ovarian cancer cells.** Results of at least three experiments are shown. **(A)** CHAC1 protein down-regulation after treatment with siRNA. **(B)** *In-vitro* Scratch Assay and Proliferation analysis of Wild-type breast cancer cells, CHAC1 knock-down cells (CHAC1-siRNA) and mock-transfected cells (scrbl-siRNA) cells. **(C)** CHAC1 protein overexpression after transfection with CHAC1-pcDNA6 or the pcDNA6 control vector. **(D)** *In-vitro* Scratch Assay and Proliferation analysis of Wild-type breast cancer cells, CHAC1 overexpressing cells (CHAC1-pcDNA6) and mock-transfected cells (pcDNA6) cells. Results of scratch assays were plotted as percentage of wound closure relative to hour 0.
